# Perforated diverticulitis presenting as necrotising fasciitis of the leg

**DOI:** 10.1186/1749-7922-3-10

**Published:** 2008-02-27

**Authors:** Timothy J Underwood, Jeremy Southgate, Robert Talbot, Guy F Nash

**Affiliations:** 1 Poole Hospital NHS Foundation Trust, Longfleet Road, Poole, BH15 2JB, UK

## Abstract

Diverticulosis of the colon is a common condition of increasing age. Complications of diverticulitis including stricture, perforation and fistula formation often require surgery. Perforated diverticulitis may rarely present with spreading superficial sepsis. We describe for the first time, to our knowledge, a case of retroperitoneal diverticula perforation presenting as necrotising fasciitis of the leg necessitating hind-quarter amputation.

## Background

Colonic diverticulosis is a common condition in the Western World and its incidence increases with advancing age [1]. Diverticula of the large bowel are believed to occur at areas of weakness, commonly at the site of entry of blood vessels as a result of increased intraluminal pressure [2]. Complications of diverticulosis include stricture, bleeding, perforation and fistula formation [1]. Perforation as a result of infected diverticulitis often leads to intra-abdominal sepsis and peritonitis requiring emergency surgery [2]. Uncommonly diverticulitis perforates into the anterior abdominal wall or retroperitoneum causing spreading infection that may require massive debridement [3,4].

Intra-abdominal sepsis may rarely present as soft tissue infection of the thigh and this has been documented as a complication of appendicitis [5]. In one case of appendicitis disarticulation of the hip was required to treat necrotising fasciitis[6]. A literature search has shown there to be no previously documented cases of diverticula perforation presenting in this way. Here we present a case of diverticulitis with retroperitoneal perforation presenting as necrotising fasciitis of the left leg requiring disarticulation of the hip.

## Case presentation

**Figure 1:**
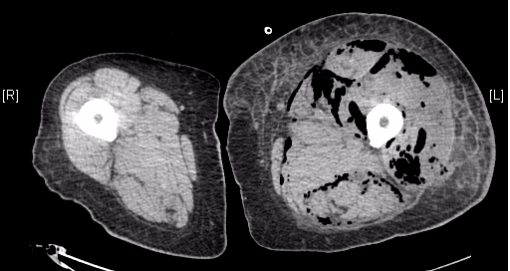
Computed tomography demonstrating gas in all muscle compartments of the left thigh.

**Figure 2:**
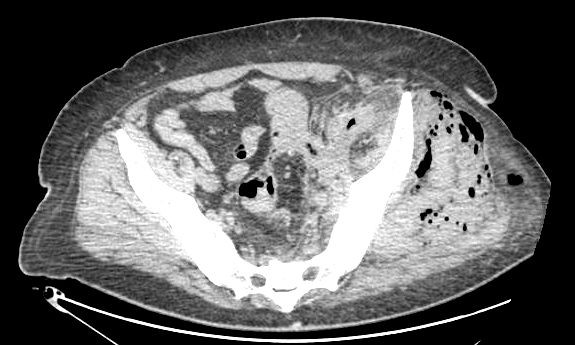
Computed tomography demonstrating gas in the left gluteal muscles and left psoas.

**Figure 3:**
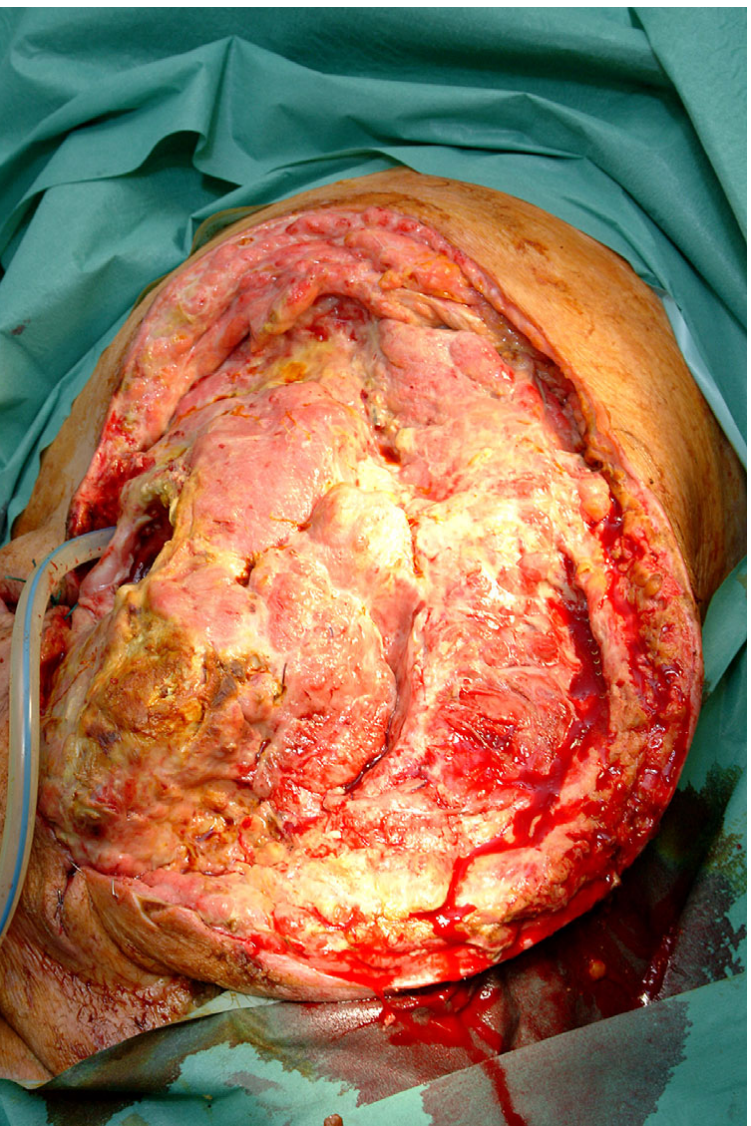
Massive open wound following left hip disarticulation and wound debridement for necrotising fasciitis prior to vac dressing.

A 51 year old man presented to the emergency department with a four day history of a painful, swollen left knee. He had previously been diagnosed with diverticulosis, in another unit, but at the time this was deemed not to require surgery. He had signs of systemic sepsis and was treated with broad spectrum antibiotics. An urgent CT of the abdomen, pelvis and legs showed a grossly enlarged left thigh with gas in all muscle compartments (Fig. 1). He was demonstrated to have gas in the gluteal muscles and gas extending along the left psoas muscle into the retroperitoneum (Fig. 2). In addition there was perinephric inflammation and the kidney was considered as the initial septic focus. A diagnosis of necrotising fasciitis was made and he was transferred to the operating theatre for surgical debridement. He required disarticulation of the left hip and excision of the gluteal muscles and part of the left psoas. A large drain was placed along the remaining psoas muscle into the retroperitoneum to allow drainage of remaining sepsis. The peritoneal cavity was opened at the time of surgery via the flank wound and the proximal sigmoid appeared normal with no evidence of intra-abdominal sepsis. Initial CT had not suggested an intra-abdominal cause of sepsis, therefore a laparotomy was not performed. His antibiotic regimen was tailored to the sensitivities of the Gram positive cocci and Gram negative bacilli cultured from tissue specimens. He underwent further minor debridement at 48 hours. At day 7 faeces appeared in the psoas drain and at formal laparotomy a segment of distal sigmoid colonic diverticulitis that had perforated into the retroperitoneum was resected by Hartmann's procedure. He recovered well enough from this surgery to allow the application of a vacuum dressing at day 17 (Fig. 3). His wound was subsequently managed by the regional plastic surgery service.

## Discussion

In the U.S an individual's risk of developing diverticulosis approaches 50% by the age of 60 [1]. Diverticulitis, infection and inflammation as a consequence of diverticula, occurs in 20–30% of patients with diverticulosis [1]. Perforation is a well recognised complication of diverticulitis often requiring emergency surgery. However, necrotising fasciitis as a consequence of perforated diverticulosis is an uncommon but potentially lethal condition requiring prompt surgical intervention [4]. Previous reports have documented the presentation of inta-abdominal sepsis either as a consequence of diverticulitis or acute appendicitis in the retroperitoneal space [3,5,6]. We have described for the first time, to our knowledge, a case of perforated sigmoid diverticulitis that led to significant retroperitoneal infection and subsequent tracking of infection via the left psoas into the left thigh, necessitating hip disarticulation.

The patient had been assessed previously for diverticulosis but surgery was not considered at that time. The recent move away from resection for uncomplicated diverticular disease is supported by large series [7,8] but such septic complications may become more common as a result of this change.

## Competing interests

The author(s) declare that they have no competing interests.

## Authors' contributions

JS, GFN, RT and TJU were responsible for the surgery and ongoing care of the patient in hospital. TJU and GFN conceived of the case report. TJU drafted the manuscript. All authors read and approved the final manuscript.

## Consent

Written informed consent was obtained from the patient for publication of this case report and any accompanying images. A copy of the written consent is available for review by the Editor-in-Chief of this journal.
